# Seasonal and Morphology Effects on Bioactive Compounds, Antioxidant Capacity, and Sugars Profile of Black Carrot (*Daucus carota* ssp. *sativus* var. *atrorubens* Alef.)

**DOI:** 10.3390/foods13101575

**Published:** 2024-05-18

**Authors:** José Luis Ordóñez-Díaz, Isabel Velasco-Ruiz, Cristina Velasco-Tejero, Gema Pereira-Caro, José Manuel Moreno-Rojas

**Affiliations:** 1 Department of Agroindustry and Food Quality, Andalusian Institute of Agricultural and Fisheries Research and Training (IFAPA), Alameda del Obispo, Avda. Menéndez-Pidal, 14004 Córdoba, Spain; josel.ordonez@juntadeandalucia.es (J.L.O.-D.); mariag.pereira@juntadeandalucia.es (G.P.-C.); 2 Departamento de Bromatología y Tecnología de los Alimentos, Campus Rabanales, Ed. Darwin-Anexo Universidad de Córdoba, 14071 Córdoba, Spain

**Keywords:** black carrot, antioxidant capacity, flavonoids, anthocyanins, simple sugars, carotenoids, HPLC-MS, vegetables

## Abstract

Black carrot (*Daucus carota* ssp. *sativus* var. *atrorubens* Alef.) is widely recognized for its bioactive compounds and antioxidant properties. The black carrot of Cuevas Bajas (Málaga) is a local variety characterized by a black/purple core, which differs from other black carrot varieties. Therefore, this autochthonous variety was characterized according to the root size and the harvesting season by means of a study of its antioxidant capacity analyzed by three methods, its total carotenoids content, and its sugars and phenolic compounds profile by ultra-high performance liquid chromatography coupled to high-resolution mass spectrometry (UHPLC-MS). A total of 20 polyphenolic compounds were quantified in 144 samples analyzed. The anthocyanidins group was observed to be the most abundant, followed by the hydroxycinnamic acids group. Moreover, pelargonidin 3-sambubioside was observed in black carrot for the first time. The medium-sized carrots presented the highest content of phenolic compounds, largely due to their significantly higher anthocyanidins content. Comparatively, the small carrots showed a higher content of simple sugars than the large ones. Regarding the influence of season, significantly higher quantities of glucose and fructose were observed in the late-season carrots, while sucrose was the main sugar in early-season samples. No significant differences were observed in the total carotenoid content of black carrot.

## 1. Introduction

The last few decades have seen a paradigm shift from the consumption of processed foods to natural, healthier food products [1]. One reason for this trend is that foods like fruits and vegetables are a good source of an extensive range of phytochemicals with proven health-promoting properties. Among them, black carrot (*Daucus carota* ssp. *sativus* var. *atrorubens* Alef.) has presented a higher antioxidant capacity and quantity of polyphenols than other vegetables, including the more widely consumed orange carrot [2,3]. Black carrot originated in Middle Asia, although the consumption of its different varieties has been increasing in Western European countries in the last decade [4].

The phytochemical content of black carrot differs greatly from other colored carrots, with them being a richer source of phenolic compounds [3,5]. The polyphenol profile of black carrots is remarkable for a high content of anthocyanins, a group of water-soluble pigments responsible for the purple color in many fruits and vegetables [6]. Anthocyanins constitute the largest group of bioactive compounds in black carrots, with acylated compounds being the major anthocyanin derivatives present in this vegetable [5]. Therefore, black carrot anthocyanins have been the focus of numerous investigations [7,8]. The literature reports high contents of hydroxycinnamic acids and flavonoids such as chlorogenic acid, caffeic acid, ferulic acid, and epicatechin [8]. This characteristic polyphenolic profile is important in terms of pharmacological properties, as it is related to its antioxidant, antitumor, and anti-inflammatory activities, and to preventing the effects of metabolic disorders [9]. In addition, products derived from black carrots [10,11] or their use to enrich other products [12,13] have been widely evaluated, promoting their potential application in the food industry.

However, the phytochemical composition of black carrot depends on many factors, such as cultivar, root size, soil, irrigation, and the environmental conditions during production [14,15].

Specifically, black carrot, known in Spain as “zanahoria morá”, is an indigenous crop from Cuevas Bajas with distinctive features that are specific to the production area, such as its roots with a purple core, which positively influence its polyphenolic profile. The cultivation of this autochthonous variety is limited to small farms in an endangered area. The promotion of its cultivation is an opportunity for rural development and helps to prevent the depopulation of the area, objectives within the common agricultural policy (CAP) of the European Commission. Moreover, black carrots traditionally have been harvested in November, December, and January. This early harvesting greatly limits the production capacity of the location. Extending the harvesting periods to February, March, and April would increase the economic value of this crop.

This study aims to chemically characterize the black carrot from Cuevas Bajas and to investigate its polyphenol profile, antioxidant activity, and individual sugar and total carotenoid content. Therefore, this study will provide information about how the phenolic profile of the zanahoria morá varies according to the growing season and the root size at harvesting time.

## 2. Materials and Methods

### 2.1. Chemicals

The reference compounds 3,4-dihydroxycinnamic acid, chlorogenic acid, 4-hydroxy-3-methoxycinnamic acid, 4′-hydroxycinnamic acid, 3,4,5-trihydroxycinnamic acid, 4-hydroxybenzoic acid, (−-epicatechin, apigenin, cyanidin-3-glucoside, (±)-6-hydroxy-2,5,7,8-tetramethylchromane-2-carboxylic acid (Trolox), and β-carotene, and the individual sugars glucose, fructose, and sucrose, were obtained from Sigma-Aldrich (Steinheim, Germany). Formic acid was obtained from Sigma-Aldrich. LC-MS grade water, methanol, ethanol, dichloromethane, and acetonitrile were acquired from PanReac Applichem (Castellar del Vallés, Barcelona, Spain).

Note: the names for the phenolic compounds used in this paper are based on the nomenclature thesaurus of Kay et al., 2020 [16].

The reagents 2,2′-azino-bis(3-ethylbenzothiazoline-6-sulfonic acid) diammonium salt (ABTS), 2,2-diphenyl-1-(2,4,6-trinitrophenyl)hydrazyl (DPPH), 2,2′-azobis(2-methylpropionamidine) dihydrochloride (AAPH), fluorescein, potassium persulfate, sodium hydroxyde, potassium dihydrogen phosphate, and sodium hydrogen phosphate were also acquired from Sigma-Aldrich (Steinheim, Germany).

### 2.2. Materials, Sample Collection, and Preparation

The black carrot samples were grown in a Mediterranean cultivation area. These soils are free-draining, highly porous, and have a very abundant mineral composition characteristic of the area. No chemical fertilizers were used. The samples were harvested in two consecutive harvesting seasons: early-season (from November to January) and late-season (from February to April) over two years 2016–17 and 2017–18 from Cuevas Bajas (Málaga, Spain). The weather conditions of both periods are detailed in Table S1 [17]. The early-season carrots were planted at the end of August and harvested at a crop age of 3–5 months (from November to January), while late-season carrots were planted in October and harvested from February to April.

Upon arrival in the laboratory, the black carrots were classified into different independent groups by root size: small (<30 mm diameter), medium (between 30 and 60 mm diameter), and large (>60 mm diameter). Thus, a total of 144 black carrot samples were organized according to carrot size into 36 groups, namely 8 big, 10 medium, and 18 small sizes, each group consisting of 4 carrots. Besides, the samples were also classified as early-season (n = 25) and late-season (n = 11) black carrots.

The black carrot samples from each group were cut and ground with a Sammic Cutter SK-3 and subsequently lyophilized using a Labconco Stoppering Tray Dryers lyophilizer, and then ground to a final particle size of 10 μm using a mixer mill (Retsch GmbH, Haan, Germany). They were then stored at –80 °C until their extraction and analysis.

For polyphenol analysis and assays of antioxidant activity, the black carrot samples were extracted adapting a previously published protocol optimized by Moreno-Rojas et al. [15]. Briefly, 0.2 g of lyophilized and ground sample was mixed with 1 mL of deionized water:methanol (80:20, *v*/*v*) with 1% formic acid for 2 min and the mixture was sonicated for 15 min and then centrifuged at 15,000 rpm for 20 min at 4 °C. The supernatant was collected and the residue was re-extracted twice using 1 mL of the same solvent following the same protocol described previously. All supernatants were pooled to a final volume of 2 mL and frozen at −80 °C until their analysis. Sugars were extracted from the black carrot samples following the method previously described by Moreno-Ortega et al. [18]. Briefly, 0.2 g of sample was mixed with 1 mL of deionized water:ethanol (20:80, *v*/*v*), sonicated in ultrasonic bath for 10 min, and centrifugated at 15,000 rpm for 15 min. The supernatant was collected and the residue was re-extracted twice using 1 mL of the same solvent following the same protocol described previously. All the supernatants were pooled together and frozen at −80 °C until HPLC-RID analysis.

### 2.3. Analysis of Flavonoids, Phenolic Acids, and Anthocyanins

The identification and quantification of flavonoids and anthocyanins in the black carrot extracts were carried out using a UHPLC-HR-MS mass spectrometer system (Thermo Scientific, San José, CA, USA) consisting of a UHPLC pump, a PDA detector scanning from 200 to 600 nm, an autosampler operating at 4 °C (Dionex Ultimate 3000 RS, (ThermoFisher Scientific, San Jose, CA, USA), and an Exactive Orbitrap mass spectrometer.

#### 2.3.1. Analysis of Flavonoids and Phenolic Acids

The analysis of phenolic compounds was based on the protocol previously described by Pereira-Caro et al. [11]. The separation was performed on a Kinetex C18 (150 mm × 4.6 mm; i.d., 5 μm 100 A (Phenomenex, Macclesfield, UK) preceded by a guard pre-column of the same stationary phase and maintained at 40 °C. An Exactive Orbitrap mass spectrometer (Thermo Scientific, San José, CA, USA) fitted with a heated electrospray ionization probe (HESI) was used to determine the polyphenols. The operating method was in negative ionization mode.

Quality control samples (QC) were applied to assess and ensure the analytical process. Data acquisition and processing were carried out using Xcalibur 3.0 software (Thermo Scientific, San José, CA, USA).

#### 2.3.2. Analysis of Anthocyanins

The anthocyanins in the black carrot extracts were separated following the protocol previously described by Ordoñez-Díaz et al. [19]. Subsequently, the eluate from the column was directed straight to an Exactive Orbitrap mass spectrometer (Thermo Scientific, San José, CA, USA), equipped with a heated electrospray ionization probe (HESI), functioning in positive ionization mode to ascertain the presence of anthocyanins. 

#### 2.3.3. Identification and Quantification of Flavonoids and Anthocyanins

Targeted identifications of polyphenols were achieved as follows: (i) by comparing the exact mass and the retention time with available standards; and (ii) in the absence of standards, compounds were tentatively identified by comparing the theoretical exact mass of the molecular ion with the measured accurate mass of the molecular ion, and a search was performed of metabolite databases such as Metlin, Phenol Explorer, and more general chemical databases including PubChem and ChemSpider.

Metabolites with molecular masses falling within the predetermined tolerance (≤5 ppm) of the target masses were obtained from these databases. Polyphenols were measured by identifying the theoretical exact mass of the molecular ion using standard curves. If reference compounds were unavailable, quantification was based on the calibration curve of a closely related parent compound.

### 2.4. Analysis of Simple Sugars

The quantification of glucose, fructose, and sucrose in the black carrot extracts was carried out using the method described by Moreno-Ortega et al. [18]. The sugars were identified by comparing the retention times with pure reference standards. Quantification was performed by reference to the 0.3–50.0 g/L calibration curves of fructose and 0.3–10.0 g/L of glucose and sucrose.

### 2.5. Analysis of Antioxidant Activity

#### 2.5.1. ABTS Assay

The free radical scavenging activity was measured using the ABTS decoloration method by Ordoñez-Diaz et al. [19]. The antioxidant activity was represented as mg of Trolox equivalents per 100 g of dry sample (mg TE/100 g DW). Values represent the mean of three measurements.

#### 2.5.2. DPPH Assay

Free radical DPPH (1,1-diphenyl-2-picryl-hydrazyl) scavenging capacity was determined using the method previously described by Pereira-Caro et al. [11]. The antioxidant activity was expressed as mg of Trolox equivalents per 100 g of dry sample (mg TE/100 g DW). Each value is the average of three measurements.

#### 2.5.3. ORAC Assay

The oxygen radical scavenging activity was measured by the ORAC assay, according to the method previously published by Tuárez-García et al. [20]. ORAC values are expressed as mg of Trolox equivalents per 100 g of dry sample (mg TE/100 g DW).

### 2.6. Analysis of Total Carotenoids

The lipophilic fraction extraction and saponification were conducted according to a method previously described by Ordóñez-Díaz et al. [19], with some adjustments. Total carotenoids were quantified using the procedure outlined by Morales et al. [21]. The lipophilic extract was dissolved in dichloromethane and transferred to a flat-bottomed 96-well quartz microplate with a well capacity of 300 µL (Hellma Analytics, Plainview, NY, USA). Absorbance was measured at 450 nm using a Synergy HTX Multi-Mode Microplate Reader (Biotek Instruments, Winooski, VT, USA). Total carotenoids were expressed as mg of β-carotene equivalents per 100 g of dry sample (mg βC/100 g DW). All measurements were performed in triplicate.

### 2.7. Statistical Analysis

The data underwent analysis of variance (ANOVA) to discern variations among the samples. A two-way ANOVA was performed, followed by a means comparison using the Tukey post hoc test, conducted using R software (v. 3.6.3, R Core Team, Vienna, Austria). The level of significance was established at *p* < 0.05. The Pearson correlation (*p* ≤ 0.01) was analyzed by the OriginPro software, version 2024 (free trial version) (OriginLab Corporation, Northampton, MA, USA).

## 3. Results and Discussion

### 3.1. Effect of the Harvesting Season Period and the Root Size on Individual Sugars of Black Carrot

**Figure 1:**
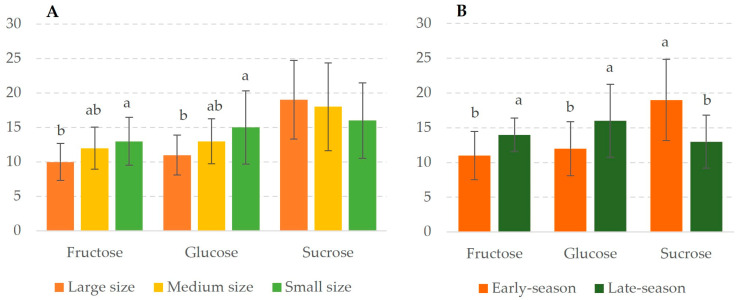
Sugar content in black carrot samples from different root sizes (**A**) and harvesting season periods (**B**). Data are expressed as g/100 g DW as mean values. Different letters (one-way ANOVA) are present to denote statistically significant differences between the different factors under study (*p*-value < 0.05).

Figure 1 shows the content of fructose, glucose, and sucrose present in black carrots obtained by HPLC-RID. Focusing on individual sugars, sucrose was the main sugar quantified in all three carrot sizes, followed by glucose (Figure 1A). Glucose, fructose, and sucrose confirm the simple sugar content of black carrot [22]. Therefore, these three sugars are responsible for the sweet taste of black carrot. The results showed significant differences between the samples by size. Small carrots presented a higher content of fructose and glucose than large carrots. This could be explained by the synthesis of fructose while the plant matures and grows [23]. Regarding the harvesting season period, significant differences were observed in the sugar profile (Figure 1B). The early-season carrots had a lower concentration of glucose and fructose and a higher content of sucrose, whilst late-season carrots contained more of the two monosaccharides and less sucrose. A reverse trend was noted between the content of fructose and glucose in carrots versus the sucrose content. According to our results, collecting small carrots early in the season could be a good strategy for increasing the naturally sweet taste.

Black carrots have a high concentration of glucose and fructose, making them sweeter than commercial orange carrots [24]. In the literature, orange carrots have reported a lower content of the monosaccharides glucose (1.13 g/100 g) and fructose (1.16 g/100 g), as well as the disaccharide sucrose (2.19 g/100 g) [24] when compared with the mean concentration obtained in these black carrot samples (13.4 g/100 g of glucose, 12 g/100 g of fructose, and 17 g/100 g of sucrose). The choice of variety seems to be the most important pre-harvest factor for the nutritional quality of carrots [14]. This makes black carrots more desirable for fresh consumption and the elaboration of derived products [25], and, therefore, of greater value to the food industry. To this end, previous studies have positively evaluated the use of black carrot powder in baked products. The results showed an increase in consumer acceptability of these healthier products [26,27]. Moreover, the local carrot studied here presented significantly higher sugar content than other black carrots found in the literature [3,24], so the addition of free sugars or sweeteners to make manufactured products would be reduced, increasing their health benefits, a feature highly demanded by consumers.

### 3.2. Identification and Quantification of Polyphenols in Black Carrot

The basis of the proposed identification is shown in Table 1. For additional information on the HPLC-HR-MS profiles, see Figures S1 and S2 in Supporting Information. A total of 20 phenolic compounds were tentatively identified and quantified in the black carrot samples, namely four hydroxybenzoic acid derivatives (3.4.5-trihydroxybenzoic acid, 3,4-dihydroxybenozic acid, 4-hydroxybenzoic acid, and 2.4-methoxy.4-hydroxybenzoic acid), four hydroxycinnamic acid derivatives (chlorogenic acid, 3,4-dihydroxycinnamic acid, 4-hydroxy-3-methoxycinnamic acid, and o-feruloylquinic acid), two flavanones (two isomers of eriodictyol-glucoside), one flavonol (quercetin-3-galactoside), and eight anthocyanins (including cyanidin-3-xylosyl-glucosyl-galactoside, cyanidin-3-xylosyl-galactoside, cyanidin-3-xylosyl-(sinapoylglucoside)-galactoside, cyanidin-3-xylosyl-(feruloylglucosyl)-galactoside, cyanidin-3-xylosyl-(coumaroylglucosyl)-galactoside, perlargonidin-3,5-diglucoside, pelargonidin-3-sambiburoside, and delphinidin-3-glucoside).

Most previous studies of black carrots focused on their anthocyanin profile, with little attention paid to the other polyphenol families [2,5]. In the literature, only one similar study of black carrots performed a complete analysis of the polyphenolic profile, to the authors’ knowledge [8]. In addition, to our knowledge, this study marks the first time that pelargonidin 3-sambubioside has been identified and quantified in black carrot. This compound has been previously identified in different berry fruits [28,29].

**Table 1:** Identification by HPLC-HR-MS of polyphenols in black carrots. For HPLC-HR-MS peaks see Figures S1 and S2 in Supporting Information.

Peaks	Compounds	Chemical Formula	[m/z]^−^ Theoretical	Error (ppm)	Retention Time (min)	MSIMI Level ^a^
	*Hydroxybenzoic acids*					
C1	3,4,5-Trihydroxybenzoic acid (gallic acid)	C_7_H_6_O_5_	169.0131	1.12	3.2	1
C2	3,4-Dihydroxybenzoic acid	C_7_H_6_O_4_	153.0182	1.60	5.9	1
C3	4-Hydroxybenzoic acid	C_7_H6O_3_	137.0243	1.82	9.3	1
C4	2,4-Methoxy-4-hydroxybenzoic acid (siringic acid)	C_9_H_10_O_5_	197.0445	4.01	16.8	1
	*Hydroxycinnamic acids*					
C5	Chlorogenic acid	C_16_H_18_O_9_	353.0867	1.63	11.7	1
C6	3,4-Dihydroxycinnamic acid (caffeic acid)	C_9_H_8_O_4_	179.0351	2.31	11.8	1
C7	4-Hydroxy-3-methoxycinnamic acid (ferulic acid)	C_10_H_10_O_4_	193.0516	3.29	15.2	1
C8	*O*-Feruoylquinic acid	C_17_H_20_O_9_	367.1024	1.31	14.4	2
	*Flavanones*					
C9	Eriodictiol-*O*-glucoside (I)	C_21_H_22_O_11_	449.1078	−1.48	12.6	2
C10	Eriodictiol-*O*-glucoside (II)	C_21_H_22_O_11_	449.1078	−1.48	15.8	2
	*Flavonols*					
C11	Quercetin-3-galactoside	C_21_H_20_O_12_	463.0871	3.06	15.9	2
	*Anthocyanins*					
C12	Cyanidin-3-xylosyl-glucosyl-galactoside	C_32_H_38_O_20_	743.2039 ^b^	−2.12	5.1	2
C13	Cyanidin-3-xylosyl-galactoside	C_26_H_28_O_15_	581.1497 ^b^	-0.6	5.3	2
C14	Cyanidin-3-xylosyl(sinapoylglucosyl)galactoside	C_43_H_48_O_23_	949.2608 ^b^	−1.26	5.6	2
C15	Cyanidin-3-xylosyl(feruoylglucosyl)galactoside	C_42_H_46_O_23_	919.2503 ^b^	−2.75	5.7	2
C16	Cyanidin-3-xylosyl(coumaroylglucosyl)galactoside	C_41_H_44_O_22_	889.2397 ^b^	−0.76	5.7	2
C17	Peonidin 3-xylosylglucosylgalactoside	C_33_H_40_O_20_	757.2185 ^b^	−3.85	8.2	2
C18	Pelargonidin-3-sambiburoside	C_26_H_28_O_14_	565.1544 ^b^	−1.42	5.6	2
C19	Pelargonidin-3.5-diglucoside	C_27_H_30_O_15_	595.1641 ^b^	−0.53	5.7	2
C20	Delphinidin-3-glucoside	C_21_H_20_O_12_	465.1028 ^b^	−0.87	6.4	1

^a^ Metabolite Standards Initiative metabolite identification (MSIMI) levels [30]. Reference compounds were available for all compounds identified at MSIMI level 1. Compounds at MSIMSI level 2 were tentatively identified. ^b^ Compounds identified with positive ionization [m/z]^+^.

The mean concentration of phenolic compounds quantified in the black carrot samples, divided into three different root sizes and two harvesting season periods, is presented in Table 2. In general, a high content of phenolic compounds (340–432 mg/100 g DW) was found in the samples. This black carrot variety was found to have a higher content of phenolic acids than other black carrots reported previously in the literature [31]. Anthocyanins followed by hydroxycinnamic acids represented the major groups quantified in the samples. The contents of hydroxybenzoic acids, flavonols, and flavanones groups were significantly lower.

Black carrot is known for its high anthocyanin content due to its characteristic color. Indeed, other studies reported that black carrots have significantly larger amounts of anthocyanins compared to carrots of other colors [3,5]. Anthocyanins represented between 45.6–49.5% of the total phenolic compounds in all the carrot samples evaluated. These compounds are widely known for their health-promoting and pigmenting properties [32]. Therefore, considering that consumers are increasingly demanding more natural food processing, the use of colored foods instead of synthetic dyes would improve the nutritional quality and economic value of food products. Regarding the anthocyanins profile, cyanidin-derived compounds accounted for 99.5% of the total anthocyanin content in the black carrots evaluated, cyanidin 3-xylosyl(feruloylglucosyl)galactoside being the main compound for all the sizes and seasons. Moreover, other anthocyanin compounds (peonidin 3-xylosylglucosylgalactoside, pelargonidin 3,5-diglucoside, pelargonidin 3-sambubioside, and delphinidin 3-glucoside) were detected in lower concentrations. In general, these anthocyanin compounds present important antioxidant and anti-inflammatory properties [33,34], proving their remarkable importance in black carrot.

Among phenolic acids, hydroxycinnamic acids represented 98% of the total content, with a great contribution of o-feruloylquinic acid and chlorogenic acid (Table 2). It was noted that o-feruloylquinic acid was the main compound in black carrot, representing among the 72.8–79% of the total phenolic acids content. This information contrasts with other results from the literature, where various authors reported chlorogenic acid as the main phenolic acid in black carrot [3,8]. This is explained by the absence of feruloylquinic acid among the compounds identified in most of the articles.

Significant differences were found in the concentration of polyphenols according to the size of the black carrots. The medium ones showed the highest total phenolic content. Specifically, they presented higher contents of total anthocyanins than the small and big sizes. Focusing on their anthocyanins profile, a significantly higher content of cyanidins was found in the medium-sized carrots, with a notable contribution from the cyanidin 3-xylosyl-glucosyl-galactoside, cyanidin 3-xylosyl-galactoside, and cyanidin 3-xylosyl(feruloylglucosyl)galactoside compounds. A remarkably higher content of hydrocinnamic acids were also obtained in the medium-sized samples due to the high contribution of feruloylquinic acid and chlorogenic acid compounds.

The carrots harvested late in the season presented a significantly higher content of total phenolic compounds, due to their higher phenolic acid content. Those harvested early in the season presented a two-fold higher content of the hydroxybenzoic acids group of compounds, mainly due to the contribution of protocatechuic acid, 4-hydroxybenzoic acids, and gallic acid compounds, whilst the black carrots harvested during the late season showed a significantly higher amount of the hydroxycinnamic acids group of compounds. Regarding the anthocyanins profile, no significant differences were observed for its total content by harvesting period, but differences were observed for individual compounds. While the early-season carrots reported higher contents of cyanidin 3-xylosyl-galactoside, cyanidin 3-xylosyl(coumaroylglucosyl)galactoside, pelargonidin 3,5-diglucoside, and pelargonidin 3-sambubioside, the late-season samples showed a higher content of cyanidin 3-xylosyl-glucosyl-galactoside, cyanidin 3-xylosyl(sinapoylglucosyl)galactoside, and cyanidin 3-xylosyl(feruloylglucosyl)galactoside. It can be hypothesized that the differences found could be due to the competitive synthesis mechanism determined by the harvesting season.

### 3.3. Effect of the Harvesting Season and the Root Size on the Antioxidant Capacity of Black Carrot

**Figure 2:**
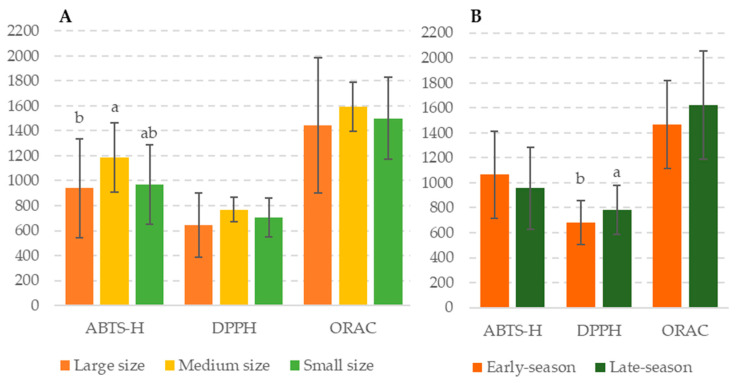
Antioxidant capacity of black carrot samples of different root sizes (**A**) and harvesting periods (**B**). Data are expressed as mg/100 g DW as mean values. Different letters are present to denote statistically significant differences (*p*-value < 0.05). ABTS-H was performed on the hydrophilic extract.

The black carrot samples analyzed from different harvesting seasons and root sizes presented high antioxidant activity values (Figure 2), according to previous research in the literature [5]. In general, the amount of total phenolic compounds determined by HPLC is linked to the antioxidant activity results obtained for root size samples (ABTS) and for harvesting season (DPPH) (Table 2). The medium-sized black carrot samples analyzed showed the highest values for antioxidant capacity using the ABTS assay. Similar trends were observed for the data obtained with the DPPH and ORAC assays, but no statistically significant differences were found for either. Regarding the harvesting periods under study, statistical differences (*p* < 0.05) were found for the DPPH method, specifically, the black carrot samples harvested during the late season showed higher antioxidant capacity values. Differences in the results of antioxidant activity obtained with the ORAC assay and those from the ABTS and DPPH assays are common due to their different chemical principles. While ORAC is based on the evaluation of peroxyl radical scavenging, ABTS and DPPH assays are based on the capacity of sample extracts to scavenge the free radical cation [35].

The black carrot samples analyzed showed much higher antioxidant capacity values (DPPH) than traditionally-consumed orange carrots (48.78 mg/100 g). Indeed, the black carrots presented higher antioxidant capacity values than any other colored carrots evaluated to date in the literature (yellow carrot: 25.47 mg/100 g; purple carrot: 545.28 mg/100 g) [24]. Anthocyanins were the main family of compounds found in the samples analyzed and have been proven to have a good free radical scavenging capacity [2,36]. These compounds have even been evaluated for their use to enrich products and increase their value [37].

### 3.4. Effect of the Season Period and the Root Size on Total Carotenoids and Lipophilic Antioxidant Capacity of Black Carrot

The total carotenoid values obtained from the samples of different sizes were not significantly different (Table 3). Moreover, significant differences were not found in the mean values obtained for total carotenoids for the samples harvested during the early- and late-season periods (4.0 and 4.1 mg/100 g DW, respectively). These results prove the high stability of the carotenoid content in black carrots. The carotenoid content results were conditioned by the color of the root [38], being significantly higher in varieties of different colors [2]. Moreover, no significant differences were observed in the antioxidant activity evaluated in the lipophilic extract for the black carrot samples based on the two factors under study: harvesting season period or root size. These results are in agreement with previous studies of black carrots that reported a less significant value for the antioxidant capacity of the lipophilic fraction [3,39,40].

### 3.5. Pearson Correlation

**Figure 3:**
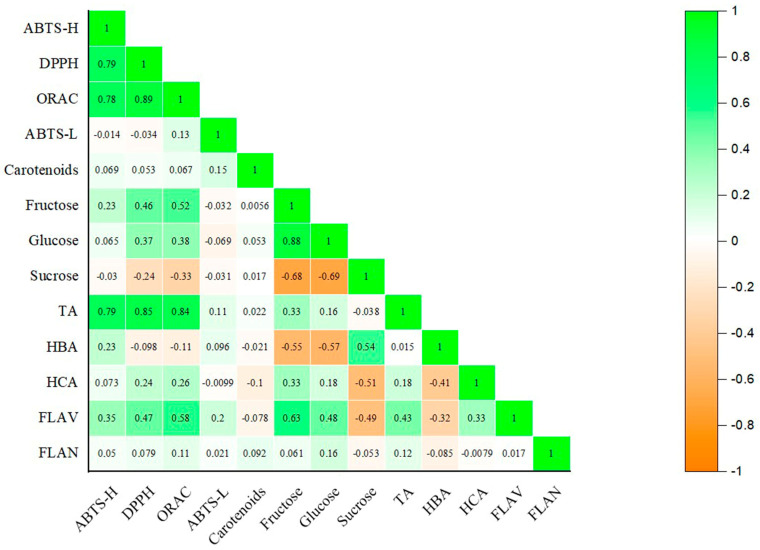
Pearson’s correlation coefficients between the antioxidant activity of the hydrophilic extract (ABTS-H, DPPH, ORAC), the total carotenoids content (TC) and antioxidant activity of the lipophilic extract (ABTS-L), the sugar content (HPLC) and total phenolic acids and flavonoids content (HPLC). TA: total anthocyanins; HBA: total hydroxybenzoic acids; HCA: hydroxycinnamic acids; FLAV: flavonols; FLAN; flavanones.

In this study, a Pearson correlation test was performed to evaluate the relationship between the different parameters evaluated (Figure 3). As expected, a high correlation was observed between the three antioxidant activity assays performed on the hydrophilic extract. In addition, a strong correlation between the antioxidant assays data and the total anthocyanins content was observed. Despite the influence of other compounds on the antioxidant capacity, this result can be explained by the fact that anthocyanins represent almost 50% of the phenolic content obtained by the chromatographic method. Moreover, this family of phenolic compounds has been widely characterized by the literature because of its great antioxidant potential [36], it being mainly responsible for the high antioxidant power of the black carrot. Meanwhile, an inverse relationship was found between monosaccharides and the disaccharide, which is evidence of the synthesis of sucrose from the glucose and fructose monosaccharides during carrot ripening. No correlation between total carotenoid content and the antioxidant activity of the lipophilic extract was found, as would be expected. This result could be explained by the contribution of other lipophilic substances present in the lipophilic extract with antioxidant capacity, such as tocopherol [18].

### 3.6. Partial Least Squares-Discriminant Analysis (PLS-DA)

**Figure 4:**
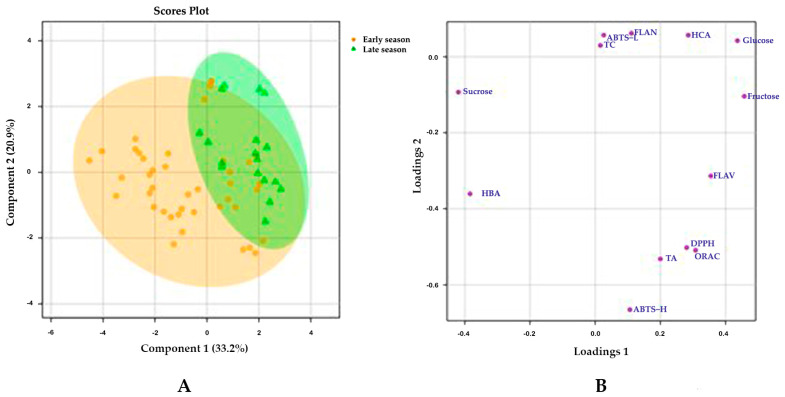
Scores plot (**A**) and loading plot (**B**) of the PLS-DA by the season period in black carrot. TA: total anthocyanins; HBA: total hydroxybenzoic acids; ABTS lip: ABTS of the lipophilic fraction; TC: total carotenoids; FLAV: flavonols; FLAN; flavanones; HCA: hydroxycinnamic acids.

Multivariate analyses (PLS-DA) were performed to evaluate the information found in this research and the factors under study. The implication of the variables in the differentiation between the samples and the degree of homogeneity within the samples of the same group were also evaluated. Figure 4 displays the differences between the samples grouped by harvesting season (early- and late-season). The subspace spanned by the first two latent variables (LV) explained 54.1% of the total variance. The first component explained 33.2% of the total variance, and the main differences between the carrot samples harvested during the early and late periods. As Figure 4B shows, the variation was mainly attributed to the sugar content and the phenolic acids profile. On the one hand, fructose and glucose, the two monosaccharides that constitute sucrose, are present in late-season carrots in higher quantities, whilst sucrose, used by maturing plants for growth, is the main sugar in the early-season carrots (Figure 1). On the other hand, the late-season carrots presented a significantly higher hydroxycinnamic acids content, whilst the early-season carrots presented a higher content of hydroxybenzoic acids (Table 2). For this reason, the phenolic acids content also contributes to the variance due to the season period.

A PLS-DA method was performed to study the main sources of variation due to the root size, but the results showed that no variable was mainly involved in the differences by size (Figure S3).

## 4. Conclusions

This study provides information about the bioactive stability of the zanahoria morá under the influence of the growing season and the root size at harvesting time. The study brought to light the strong influence of the black core root of this carrot on the anthocyanidins profile. The zanahoria morá of Cuevas Bajas presented a higher content of anthocyanidins and therefore a higher antioxidant activity. In addition, this study marks the first time that pelargonidin 3-sambubioside was observed in black carrot. Regarding the harvesting period, the late-season carrots presented a higher concentration of anthocyanins and total phenolic compounds, and a significantly higher antioxidant capacity by the DPPH method (*p* < 0.05). Other parameters studied for both harvesting periods highlight that the late season carrot could be an additional source of fresh black carrots, increasing the productivity of this crop and providing longer periods of availability of this root vegetable. These results evidence the great potential of this carrot variety, its great concentration of bioactive compounds and high sugar profile providing added nutritional value. Moreover, longer periods of availability of fresh black carrot could result in the increased production of products with health benefits, which would be of great interest to the food industry and provide an economic boost to the production area.

## Figures and Tables

**Table 2:** Concentration (mg/100 g DW) of phenolic compounds identified in black carrot samples of different root sizes (S) and harvesting seasons (H).

		Size			Season			
Compounds	Large	Medium	Small	*p-Value*	Early	Late	*p-Value*	SxH *p-Value*
3,4,5-Trihydroxybenzoic acid (gallic acid)	0.018	0.008	0.014	ns	0.019 ^a^	0.000 ^b^	**	ns
3,4-Dihydroxybenzoic acid	2.59	2.77	2.62	ns	3.22 ^a^	1.35 ^b^	***	ns
4-Hydroxybenzoic acid	1.09 ^ab^	1.17 ^a^	0.97 ^b^	*	1.15 ^a^	0.87 ^b^	***	ns
2,4-Methoxy-4-hydroxybenzoic acid (siringic acid)	0.053 ^b^	0.073 ^a^	0.066 ^ab^	*	0.067	0.06	ns	ns
*Total Hydroxybenzoic acids*	3.7	4	3.7	ns	4.5 ^a^	2.3 ^b^	***	ns
Chlorogenic acid	35	44	41	ns	40	42	ns	ns
3,4-Dihydroxycinnamic acid (caffeic acid)	0.31	0.39	0.31	ns	0.40 ^a^	0.17 ^b^	**	ns
4-Hydroxy-3-methoxycinnamic acid (ferulic acid)	4.3 ^a^	4.2 ^ab^	4.0 ^b^	*	4.2	4.0	ns	ns
*O*-Feruoylquinic acid	128	171	135	ns	130 ^b^	182 ^a^	*	ns
*Total Hydroxycinnamic acids*	167	220	181	ns	174 ^b^	228 ^a^	*	ns
*Total Phenolic Acids*	171	224	184	ns	178 ^b^	230 ^a^	*	ns
Eriodictyol-*O*-glycoside (I)	0.31 ^ab^	0.31 ^b^	0.31 ^a^	**	0.31	0.31	ns	ns
Eriodictyol-*O*-glycoside (II)	0.31	0.31	0.31	ns	0.31	0.31	ns	ns
*Total Flavanones*	0.62 ^ab^	0.61 ^b^	0.63 ^a^	*	0.62	0.62	ns	ns
Quercetin-3-O-galactoside	0.33 ^b^	0.42 ^a^	0.40 ^a^	**	0.38	0.41	ns	ns
Cyanidin 3-xylosyl-glucosyl-galactoside	10 ^b^	14 ^a^	12 ^ab^	*	10 ^b^	17 ^a^	***	ns
Cyanidin 3-xylosyl-galactoside	43 ^b^	65 ^a^	50 ^ab^	*	57 ^a^	43 ^b^	*	ns
Cyanidin 3-xylosyl(sinapoylglucosyl)galactoside	24	25	24	ns	21 ^b^	32 ^a^	***	**
Cyanidin 3-xylosyl(feruloylglucosyl)galactoside	79	91	77	ns	77 ^b^	93 ^a^	*	***
Cyanidin 3-xylosyl(coumaroylglucosyl)galactoside	11	10.4	7.8	ns	10.6 ^a^	7.1 ^b^	*	*
Peonidin 3-xylosylglucosylgalactoside	0.053	0.06	0.057	ns	0.058	0.053	ns	ns
Pelargonidin 3,5-diglucoside	0.34	0.46	0.33	ns	0.42 ^a^	0.27 ^b^	*	ns
Pelargonidin 3-sambubioside	0.19 ^ab^	0.28 ^a^	0.18 ^b^	*	0.25 ^a^	0.14 ^b^	**	ns
Delphinidin 3-glucoside	0.28 ^b^	0.48 ^a^	0.39 ^ab^	*	0.37	0.43	ns	ns
Cyanidin	167 ^b^	205 ^a^	171 ^b^	**	176	193	ns	***
Peonidin	0.053	0.060	0.057	ns	0.058	0.053	ns	ns
Pelargonidin	0.53 ^ab^	0.74 ^a^	0.50 ^b^	*	0.67 ^a^	0.40 ^b^	**	ns
Delphiinidin	0.28 ^b^	0.48 ^a^	0.39 ^ab^	*	0.37	0.43	ns	ns
*Total Anthocyanins*	168 ^b^	207 ^a^	172 ^b^	**	177	194	ns	***
*Total Phenolic Compounds*	340 ^b^	432 ^a^	357 ^ab^	**	357 ^b^	425 ^a^	**	***

Mean values with different letters in the same row present significant differences. Significant level: ns = not significant. * = *p* < 0.05. ** = *p* < 0.01. *** = *p* < 0.001. Values are expressed as means of root sizes and harvesting seasons.

**Table 3:** Total carotenoid content (mg/100 g DW) and antioxidant activity of the lipophilic extract measured by the ABTS assay of black carrot samples of different harvesting seasons (H) and root sizes (S).

		Size (S)				Season (H)		
	Large Size	Medium Size	Small Size	*p-Value*	Early-Season	Late-Season	*p-Value*	SxH *p-Value*
Carotenoids	3.9	3.9	4.2	ns	4	4.1	ns	ns
ABTS-L	27	29	25	ns	26	28	ns	ns

Significant level: ns = not significant. Data are expressed as mean values.

## Data Availability

The data presented in this study are available on request from the corresponding author due to the data are confidential.

## Supplementary Materials

The following supporting information can be downloaded at: https://www.mdpi.com/article/10.3390/foods13101575/s1. Table S1: Monthly values of the meteorological factors of the experimental period. Figure S1: Representative UHPLC-HR-MS profile of polyphenols in black carrot. Figure S2: Representative UHPLC-HR-MS profile of anthocyanins in black carrot. For peak identification see Table 1. Figure S3: Score plot (A) and loading plot (B) of the PLS-DA by root size in black carrot.
