# Supplementary material for: Seasonal and Morphology Effects on Bioactive Compounds, Antioxidant Capacity, and Sugars Profile of Black Carrot (Daucus carota ssp. sativus var. atrorubens Alef.)
Source: Foods. 2024 May 18;13(10):1575. doi: 10.3390/foods13101575 (PMC11121725; doi:10.3390/foods13101575)
Supplement: Supplementary file 1 [file foods-13-01575-s001.zip › foods-2980610-supplementary.pdf]

Table S1. The monthly values of the main meteorological factors of the experimental period (2016 to 2017 and 2017 to 2018).

Month	Temperature (°C)		Atmospheric Humidity (%)		Rainfall (mm)	
	2016 - 2017	2017 - 2018	2016 - 2017	2017 - 2018	2016 - 2017	2017 - 2018
	Min-Max	Min-Max	Min-Max	Min-Max	Accumulated	
August	18.93 - 32.62	18.34 - 33.89	26.62 - 65.97	23.64 - 71.98	0.40	40.20
September	14.28 - 30.50	13.99 - 29.45	24.84 - 71.33	25.42 - 80.05	5.20	0.00
October	12.02 - 24.99	13.10 - 26.49	37.38 - 84.88	35.00 - 82.14	25.40	48.00
November	5.26 - 16.86	5.02 - 19.70	47.86 - 96.09	39.23 - 92.89	125.20	78.00
December	4.75 - 15.35	2.31 - 14.05	53.11 - 96.80	51.65 - 98.38	78.20	38.40
January	0.06 - 13.74	1.94 - 13.75	44.70 - 97.09	57.01 - 99.82	18.00	42.00
February	5.39 - 15.68	0.24 - 13.75	51.38 - 96.99	45.48 - 97.69	75.80	24.80
March	5.36 - 18.41	5.30 - 14.75	42.21 - 94.85	53.87 - 98.66	33.40	202.80
April	8.91 - 22.37	7.76 - 19.00	33.28 - 85.53	42.34 - 96.73	8.80	75.00

Min: minimum; Max: maximum

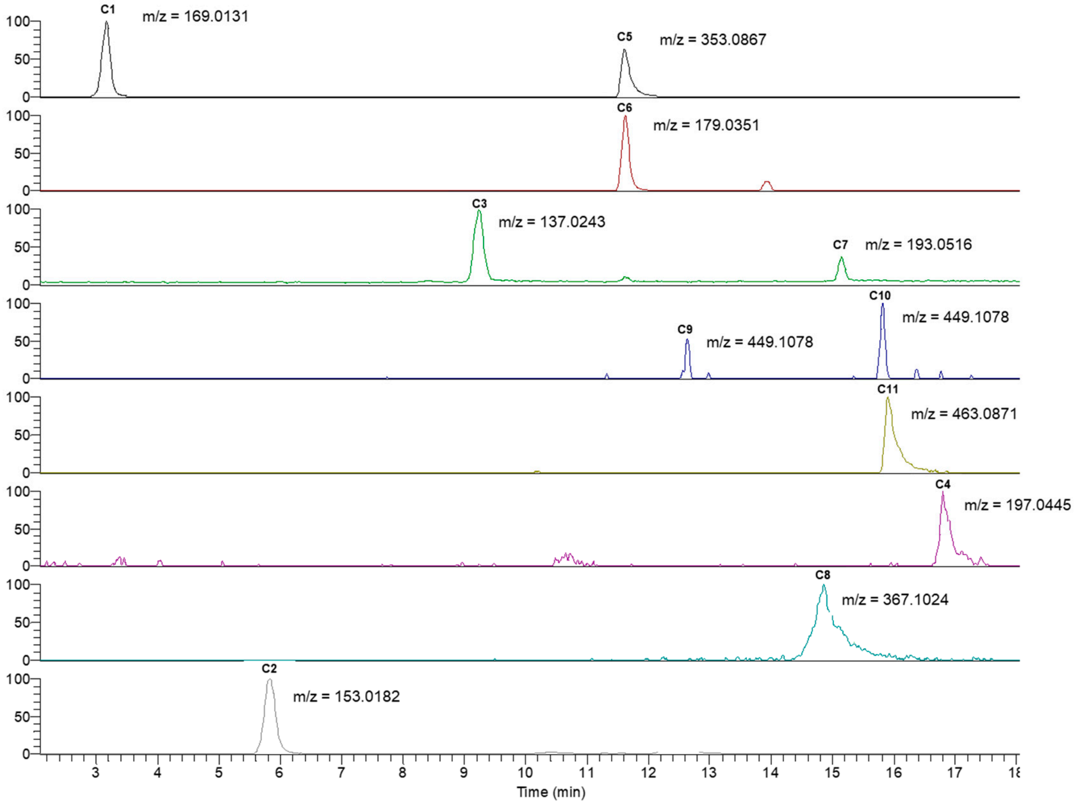

Figure S1. Representative UHPLC-HRMS profile of phenolic acids in black carrot. For peak identification see Table 1.

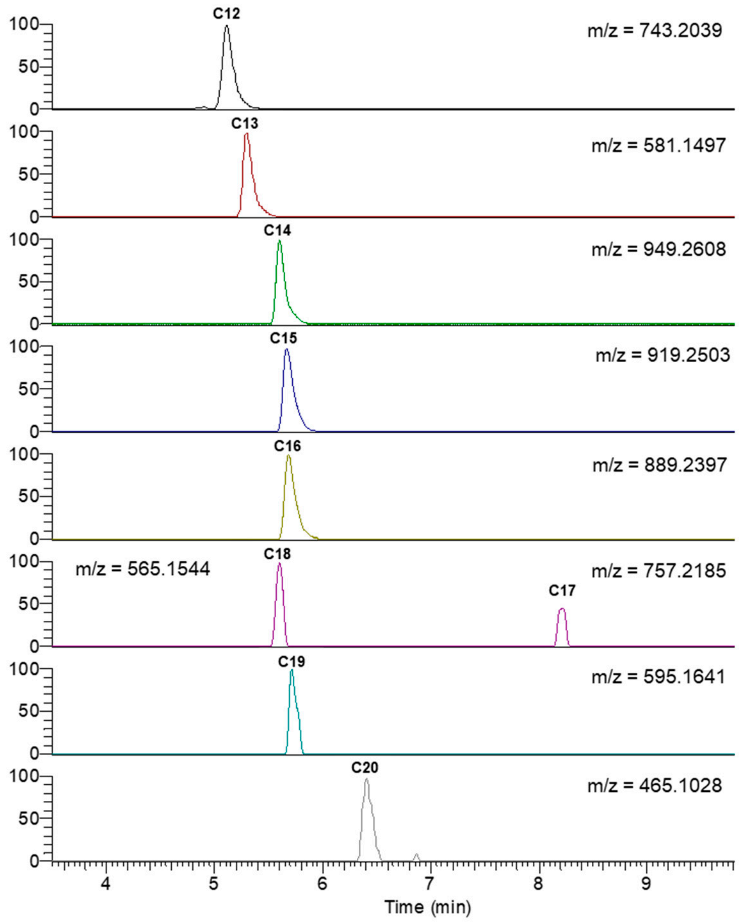

Figure S2. Representative UHPLC-HRMS profile of anthocyanins in black carrot. For peak identification see Table 1.

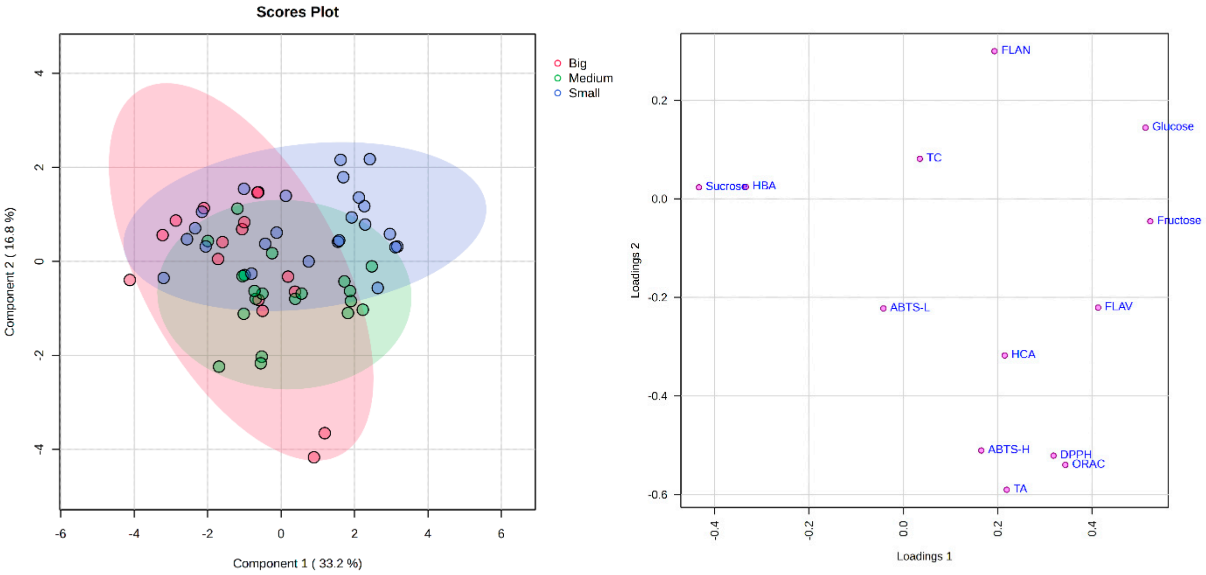

Figure S3. Scores plot (A) and loading plot (B) of the PLS-DA by the root size in black carrot. TA: total anthocyanins; HBA: total hydroxybenzoic acids; ABTS lip: ABTS of the lipophilic fraction; TC: total carotenoids; FLAV: flavonols; FLAN; flavanones; HCA: hydroxycinnamic acids.
